# In vitro antibiofilm activity of tyrosol against single and dual-species biofilms of *Candida tropicalis* and *Streptococcus mutans*

**DOI:** 10.55730/1300-0152.2779

**Published:** 2025-08-11

**Authors:** Zarifeh ADAMPOUR, Betül YILMAZ ÖZTÜRK, Bükay YENİCE GÜRSU, İlknur DAĞ

**Affiliations:** 1 Biotechnology and Biosafety Department, Institute of Science, Eskişehir Osmangazi University, Eskişehir, Turkiye; 2 Central Research Laboratory Application and Research Center, Eskişehir Osmangazi University, Eskişehir, Turkiye; 3 Vocational Health Services High School, Eskişehir Osmangazi University, Eskişehir, Turkiye

**Keywords:** Dual biofilm, *Streptococcus mutans*, *Candida tropicalis*, tyrosol, antimicrobial resistance

## Abstract

**Background/aim:**

The cross-kingdom biofilm structure formed by *Candida tropicalis* and *Streptococcus mutans* may increase caries formation. The aim of this study was to evaluate the in vitro effect of the exogenous tyrosol on single- and dual-species biofilms as well as planktonic cultures formed by *C*. *tropicalis* and *S*. *mutans*.

**Materials and methods:**

The antimicrobial efficacy of tyrosol was evaluated through broth microdilution, colony-forming unit (CFU) enumeration, and XTT reduction tests to assess cell viability and metabolic activity. Transmission electron microscopy (TEM) was used to examine ultrastructural changes in planktonic cells. Biofilm dynamics were visualized via scanning electron microscopy (SEM) and confocal laser scanning microscopy (CLSM). The in vitro cytotoxicity of tyrosol was evaluated using NIH/3T3 fibroblast cells.

**Results:**

XTT results showed that the biofilm-reducing effect of amphotericin B (AMB) on single *C*. *tropicalis* biofilm at the minimum inhibitory concentration (MIC) and 2× MIC was significantly higher than that of control (47% and 48%, respectively) (p < 0.05). Tyrosol also had a metabolic activity-reducing effect on single *C*. *tropicalis* biofilm, but this effect was not statistically significant (39% at 2× MIC and 42% at MIC). Tyrosol and ampicillin (AMP) had no significant reducing effect on single *S*. *mutans* biofilm cells (p > 0.05). However, AMP resistance increased in dual culture. CFU enumeration, TEM, SEM, and CLSM data supported these findings. The effect of tyrosol on NIH/3T3 fibroblast cells was suppressive at low concentrations (1–4 mg/mL) and enhancing at high concentrations (4.5–20 mg/mL).

**Conclusion:**

This study investigated the antimicrobial and antibiofilm properties of tyrosol against *C*. *tropicalis* and *S*. *mutans*, individually and in combination. The results showed that tyrosol inhibited growth and biofilm formation, particularly in dual-species biofilms. Although *S*. *mutans* had greater resistance, overall microbial viability was reduced. Despite some observed increase in AMP resistance, tyrosol was selectively cytotoxic, indicating its promise as a natural therapeutic agent pending further research.

## Graphical abstract

This study examined the effects of exogenous tyrosol on *Candida tropicalis* and *Streptococcus mutans* dual-species biofilms. Tyrosol reduced biofilm formation but increased ampicillin resistance. Microscopy analyses showed structural changes, highlighting the antimicrobial activity of tyrosol. These findings suggest its potential role in biofilm-associated oral infections, warranting further investigation.

**Figure:**
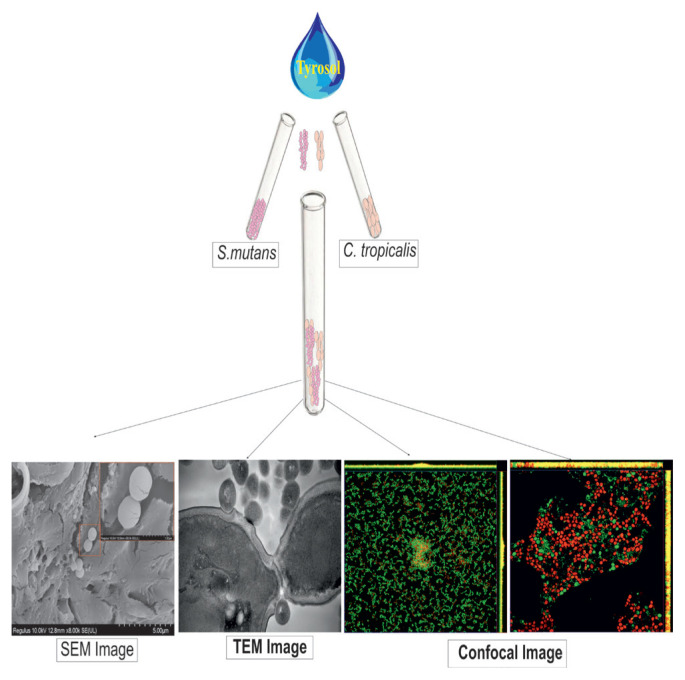


## 1. Introduction

Colonization of teeth by cariogenic bacteria is the most significant risk factor for the development of dental diseases. *Streptococcus mutans* is the primary extracellular polymeric substances (EPS) producer associated with dental caries development (Kreth et al., 2004). This species, a gram-positive facultative anaerobic bacteria, has the ability to produce acid and form amyloid, and is well adapted to biofilm formation. *S*. *mutans* can form biofilms through interkingdom polymicrobial interactions with fungal *Candida* species during the development of dental caries. This interaction increases acid production and exopolysaccharide synthesis, thereby increasing cariogenic potential (Ribeiro et al., 2012). *Candida* spp. are members of the normal microbiome and act as opportunistic pathogens, causing significant mucosal and systemic infections such as oral candidiasis in immunocompromised individuals. They are particularly found in coatings, dentine, and cement surfaces. *Candida albicans* is the most common etiological agent, followed by *C*. *tropicalis* and *C*. *glabrata* (Fumes et al., 2018). *C*. *tropicalis* is found in mixed cultures with some *Candida* species and bacteria in oral infections and can form polymicrobial biofilms. This species has true hyphal formation, increasing its virulence (Fuad et al., 2023).

The most complex pathologies related to oral diseases are caused by microbial biofilms formed by a mixed group of microorganisms and protected by a complex polymer network (Salehi et al., 2020). Studies on combating *S*. *mutans*–*Candida* spp. dual-species biofilms in the mouth have focused intensively on *C*. *albicans*. The GtfB protein of *S*. *mutans* binds to the cell wall mannoproteins in *C*. *albicans*, resulting in increased matrix synthesis and biofilm formation. The symbiotic relationship between these two species leads to the upregulation of genes related to acid production and acid tolerance. Despite *Candida* species other than *C*. *albicans*, such as *C*. *tropicalis*, are frequently found in oral infections and caries lesions, their specific role in cariogenesis progression as a result of their interaction with *S*. *mutans* remain unclear (Barbosa et al., 2016). Since the cell wall composition and adhesion properties may differ in other *Candida* species, this could influence interactions in interspecies biofilms. Thus, comprehensive studies on this topic are needed.

A biofilm matrix provides a protective niche, increasing microbial resistance to antimicrobials and facilitating chronic infections. Therefore, reliable antibiofilm strategies and effective diagnostic tools are urgently needed (Mi et al., 2018). Advanced microscopic techniques such as scanning electron microscopy (SEM), confocal laser scanning microscopy (CLSM), and atomic force microscopy offer insights into biofilm structure and treatment effects (Hung et al., 2013; Azeredo et al., 2017). In particular, CLSM allows detailed visualization of biofilm architecture using fluorescent labeling, while SEM enables surface morphology assessment, including EPS detection.

Quorum sensing (QS) has emerged as a promising strategy to interfere with biofilm development. Signaling molecules modulate gene expression in response to cell density, thereby regulating microbial physiology, including virulence and biofilm formation. Tyrosol, a QS molecule secreted by *C*. *albicans*, induces morphogenesis in a concentration-dependent manner (Cordeiro et al., 2015). Exogenous application of tyrosol can inhibit planktonic and biofilm *Candida* cells, especially when combined with antifungals (Arias et al., 2016; Uzundağ et al., 2020). However, limited information exists regarding its impact on dual-species biofilms.

This study aimed to investigate the antibiofilm effects of tyrosol on single- and dual-species models of *C*. *tropicalis* and *S*. *mutans* using microbiological, ultrastructural, confocal, and cytotoxic assays.

## 2. Materials and methods

### 2.1. Strains, cultural conditions, and antimicrobial susceptibility tests

*C*. *tropicalis* 1678 isolate was obtained from Eskişehir Osmangazi University Health, Practice and Research Hospital, Department of Microbiology. *S*. *mutans* ATCC 25175 strain was obtained commercially. The yeast strain was activated in yeast extract peptone dextrose medium at 37 °C and then incubated in Roswell Park Memorial Institute (RPMI) 1640 broth at 37 °C for 24 h. The cultures were suspended in 0.85% physiological saline and adjusted to 0.5 McFarland standard (1–5 × 10^6^ cells/mL). The suspensions were diluted 1:50 with physiological saline and 1:20 with RPMI to reach 1–5 × 10^3^ cells/mL, in accordance with the Clinical and Laboratory Standards Institute (CLSI M27-A3, 2008) guidelines.

*S*. *mutans* ATCC 25175 was obtained from the American Type Culture Collection (ATCC, Manassas, VA, USA) and cultured on brain heart infusion agar (BHI) at 37 °C in 5% CO_2_ (Zhou et al., 2025). Colonies were transferred to BHI broth and incubated statically for 18 h at 37 °C under 5% CO_2_. Cells were centrifuged at 6500 rpm for 5 min, washed with phosphate-buffered saline (PBS), and adjusted to the McFarland 0.5 standard, which corresponds to approximately 1.5 × 10^8^ CFU/mL, in accordance with the Clinical and Laboratory Standards Institute (CLSI M07-A8, 2009) guidelines, and as described by Arias et al. (2016).

For *C*. *tropicalis*, tyrosol (Sigma-Aldrich Chemical Co., St. Louis, MO, USA) was tested in concentrations from 600 to 1.17 μg/mL, prepared in sterile distilled water. A 100 μL aliquot of medium and 100 μL of serially diluted tyrosol were added to 96-well microplates. Fungal suspensions were added except in column 11 (sterility control). Amphotericin B (Sigma, USA) was tested in 16–0.0313 μg/mL concentrations. Minimum inhibitory concentration (MIC) values were determined as the lowest concentrations showing no visible growth after 24 h at 37 °C. Each test was performed in triplicate according to the CLSI M27-A3 guidelines.

For *S*. *mutans* ATCC 25175, tyrosol was tested in the 20–0.039 mg/mL range. Procedures were the same as the yeast protocol, and ampicillin (AMP) was used as a control antibiotic (330–0.31 μg/mL), following the CLSI M07-A8 guidelines. For dual cultures, isolates were coincubated in 50:50 RPMI and BHI broth. In each well, 50 μL of each cell suspension and 100 μL of tyrosol (10–0.019 mg/mL) were added. Amphotericin B (AMB) and AMP were tested again in relevant concentration ranges. All assays were performed in triplicate (Öztürk et al., 2022).

### 2.2. Transmission electron microscopy analysis of planktonic cells

Morphological effects of tyrosol on planktonic cells were examined using sterile 6-well plates. After inoculation and tyrosol treatment (MIC and 1/2 MIC), cultures were incubated at 37 °C for 24 h. Cells were fixed in 2.5% glutaraldehyde, postfixed with osmium tetroxide (OsO_4_), embedded in agar, and processed with standard TEM protocols, including uranyl acetate and lead citrate staining. Sections (60 nm) were analyzed with a Hitachi (Tokyo, Japan) HT7800 TEM. Dual cultures followed the same protocol using 50:50 RPMI and BHI medium (Öztürk et al., 2020).

### 2.3. Scanning electron microscopy analysis of biofilms

Biofilms were formed on UV-sterilized acrylic resin disks (8 × 4 mm). Acrylic resin was preferred because it is frequently used in the literature to investigate biofilm binding and structure, and because it is reproducible and easier to measure (Takamiya et al., 2021). Tyrosol was applied at MIC and 2× MIC before (prebiofilm) and after (postbiofilm) biofilm formation. Controls included no treatment, AMB, AMP, and chlorhexidine gluconate. Disks were fixed in glutaraldehyde, postfixed with OsO_4_, dehydrated in ethanol gradients, coated with gold–palladium, and examined with Hitachi Regulus 8230 SEM (Yapıcı et al., 2021).

### 2.4. XTT assay for biofilm metabolic activity

Biofilms were formed in 96-well plates. After treatment with tyrosol and control agents at MIC and 2× MIC, wells were incubated for 24 h. XTT and menadione solutions were prepared and added, and plates were incubated for 2 h at 37 °C. Absorbance was measured at 490–630 nm using a Chromate microplate reader (Molecular Devices, San Jose, CA, USA) (Ramage et al., 2002).

### 2.5. CFU quantification of biofilms

Biofilms were washed with PBS and sonicated at 30 W for 30 s. Suspensions were serially diluted and plated on Sabouraud dextrose agar and BHI for monocultures, and BHI + CHROM agar with AMB for dual cultures. CFUs were counted after 24 h at 37 °C and expressed as Log_10_ CFU/cm^2^ (Fernandes et al., 2016).

### 2.6. Hydrophobicity assay

Cell surface hydrophobicity was assessed using the sessile drop method. Polylysine-coated slides were inoculated and biofilms were treated with tyrosol or control drugs at 2× MIC. After 24 h incubation, contact angles were measured with an Attension Theta instrument (Biolin Scientific, Frölunda, Sweden) for 10 s after applying 10 μL deionized water (Arias et al., 2016).

### 2.7. Confocal laser scanning microscopy analysis

Biofilms grown in 24-well plates were treated with tyrosol at MIC and 2× MIC. LIVE/DEAD BacLight dye (SYTO 9 and PI) was applied, and samples were imaged with ZEISS (Oberkochen, Germany) LSM 800 CLSM at 40× magnification (Fernandes et al., 2018; Guo et al., 2023).

### 2.8. In vitro cytotoxicity assay

The NIH/3T3 mouse fibroblast cell line, derived from Swiss albino mouse embryo tissue, was used to evaluate the in vitro cytotoxicity of tyrosol. Mouse 3T3 fibroblasts were cultured in Dulbecco’s modified Eagle medium (DMEM)-high glucose with 10% fetal bovine serum and maintained at 37 °C in a humidified atmosphere with 5% CO_2_ (Kusena et al., 2021). Cells were seeded in 96-well plates and exposed to tyrosol (1–20 mg/mL) for 22 h. WST-1 solution was added (10% of well volume), incubated for 2 h, and absorbance was read at 450/630 nm (Park and Xian, 2015). Viability was calculated as:


$$\%\;\text{Viability}=\frac{\left(Absorbance\;of\;the\;sample\;elaute-Blank\right)}{\left(Pozitive\;Control-Blank\right)}\times 100$$

### 2.9. Statistical Analysis

Statistical evaluation of the XTT assay results was conducted using data presented as mean ± standard deviation (SD) from three independent experiments, each performed in triplicate. Prior to analysis, the assumptions of normal distribution and equal variances were assessed with the Shapiro–Wilk and Levene’s tests, respectively. Once these assumptions were met, differences among control and experimental groups were analyzed using a 1-way analysis of variance (ANOVA) test, followed by Tukey’s honestly significant difference (HSD) post hoc test for pairwise comparisons. Statistical significance was defined as p < 0.05. All analyses were performed using SPSS version 26 software.

## 3. Results

### 3.1. Antimicrobial susceptibility tests

The MIC values of tyrosol against *S*. *mutans* ATCC 25175, *C*. *tropicalis* 1678, and dual culture are presented in Table 1. AMB is much more effective than tyrosol on the *C*. *tropicalis* 1678 isolate. The single *C*. *tropicalis* 1678 culture was more sensitive to tyrosol than *S*. *mutans* ATCC 25175. For the dual culture, the MIC value of tyrosol was 2.5 mg/mL, and while this was greater than the MIC for *C*. *tropicalis* 1678 alone, it was equal to the MIC for *S*. *mutans* ATCC 25175 alone. The MIC value of AMB in the dual culture was 0.125 μg/mL—8 times lower than the effect in *C*. *tropicalis* 1678 alone. The MIC for AMP in the dual culture was 37.5 μg/mL—30 times higher than the effect on the *S*. *mutans* ATCC 25175 isolate alone.

### 3.2. TEM results for single cultures

**Figure 1:**
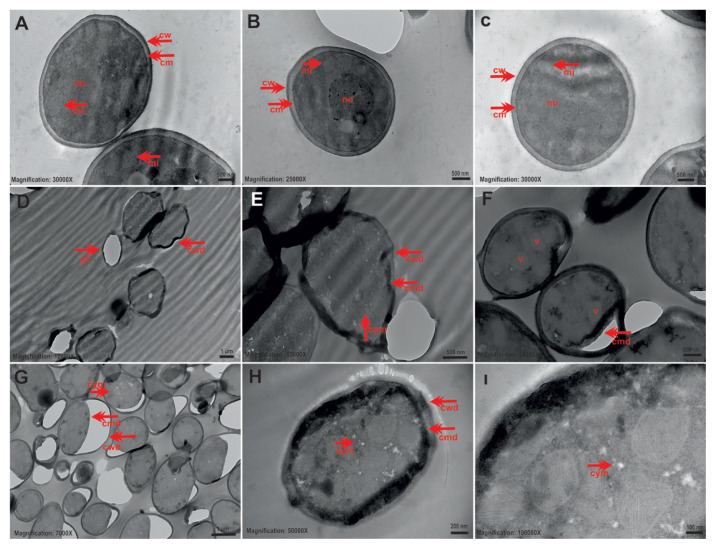
Representative TEM images showing ultrastructural changes in *C*. *tropicalis* 1678 cells before and after exposure to tyrosol. (A–C) Control group cells having intact morphology, including a healthy cell wall (cw), cell membrane (cm), nucleus (nu), and mitochondria (mi). (D–F) Cells exposed to tyrosol at 1/2 MIC showing cell wall damage (cwd), membrane disruption (cmd), ghost cells (gc), cytoplasmic melting (cym), vacuole formation (v), and separation between membrane and cytoplasm. (G–I) Cells treated with tyrosol at MIC displaying severe cytoplasmic membrane damage (cmd), ghost cell formation (gc), and cytoplasmic disintegration (cym). Scale bars: A, B, C = 500 nm; D = 1 μm; E, F = 500 nm; G = 2 μm; H, I = 200 nm.

TEM findings show ultrastructural changes between control and tyrosol application groups. Control planktonic *C*. *tropicalis* cells without tyrosol had typical and healthy *Candida* morphology. The nucleus was clearly and centrally located, the cytoplasm was regular, and the cell wall and cytoplasmic membrane structure were observed as a whole (Figures 1A–1C). In cells exposed to tyrosol at 1/2 MIC, the cell wall and membrane were folded and disordered, and there was cytoplasmic damage, increased electron density in the cytoplasm, ghost cells, cytoplasmic melts, vacuole formations, and membrane and cytoplasm separation (Figures 1D–1F). In cells exposed to the MIC of tyrosol, increased cytoplasmic membrane separations, ghost cells, cytoplasmic melts, and mitochondria damage were observed (Figures 1G–1I).

**Figure 2:**
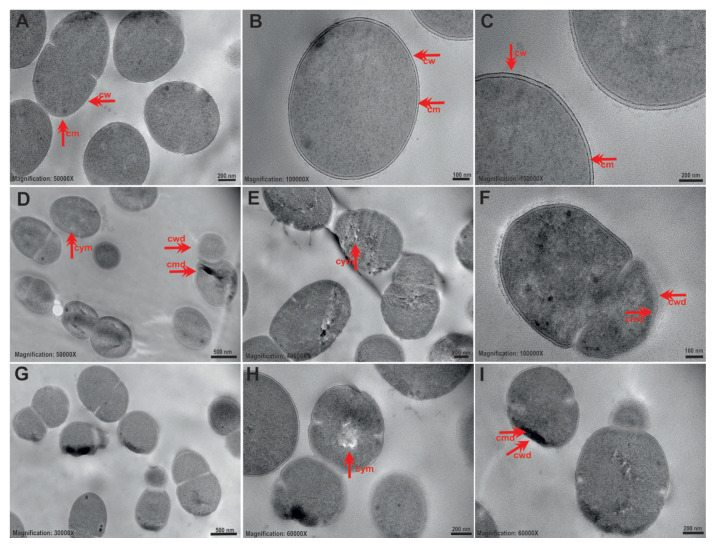
Representative TEM images showing ultrastructural alterations in *S*. *mutans* ATCC 25175 cells before and after tyrosol exposure. (A–C) Control group cells having intact morphology, including well-defined cell walls (cw) and cell membranes (cm). (D–F) Cells treated with tyrosol at 1/2 MIC displaying partial cell wall damage (cwd), membrane disruption (cmd), and cytoplasmic lysis (cym). (G–I) Cells exposed to tyrosol at MIC showing extensive membrane and cell wall damage, along with prominent cytoplasmic melting (cym). Scale bars: A, B = 200 nm; C = 100 nm; D = 500 nm; E = 200 nm; F = 100 nm; G = 500 nm; H, I = 200 nm.

The control group planktonic *S*. *mutans* ATCC 25175 cells had a typical and healthy bacterial morphology (Figures 2A–2C). The cytoplasm was homogeneously distributed and regular, and the cell wall and cytoplasmic membrane structure were intact. In cells exposed to tyrosol at 1/2 MIC, cell wall and membrane melting was observed in some places in addition to microbody (peroxisome) formation, but no deformations were observed in the cell and the cytoplasm remained relatively homogeneous (Figures 2D–2F). Membrane and wall damage, membrane folding, melting of the stopping center in a small number of cells, and microbodies were detected in cells exposed to the MIC of tyrosol (Figures 2G–2I).

### 3.3. TEM results for dual cultures

**Figure 3:**
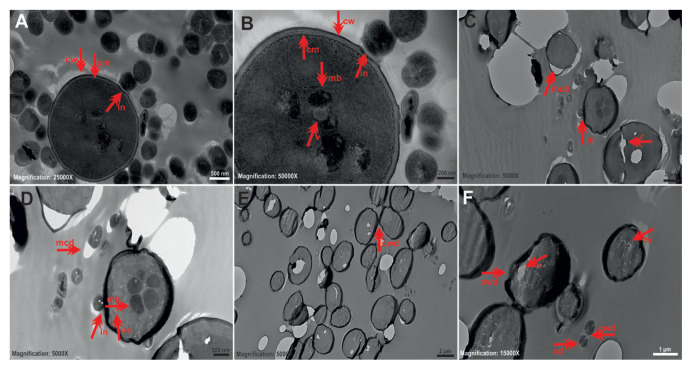
Representative TEM images showing ultrastructural changes in dual-species planktonic cultures of *S*. *mutans* ATCC 25175 and *C*. *tropicalis* 1678 before and after exposure to tyrosol. In the control group (A, B), yeast and bacterial cells have regular and oval-shaped cell walls (cw) and intact membranes (cm), with interaction regions observed between bacteria and yeast (in), a small number of microbodies (mb), and vacuoles (v) in the yeast cytoplasm. Following exposure to tyrosol at MIC (C, D), cells displayed cytoplasmic ruptures (r), prominent and numerous microbodies (mb), membrane–cytoplasm detachments (mcd), collapse-like damage in cell walls (cwd), and formation of numerous small vesicles (ve). In *S*. *mutans* cells, membrane–cytoplasm separation (mcd) and bacteria–yeast interactions (in) were noted. Upon exposure to 1/2 MIC (E, F), *C*. *tropicalis* cells showed wall collapse (cwd), cytoplasmic ruptures (r), and melting (m); whereas *S*. *mutans* cells had limited wall (cwd) and cytoplasmic damage (cd). Scale bars: A = 500 nm; B = 200 nm; C = 2 μm; D = 500 nm; E = 2 μm; F = 1 μm.

In the TEM micrographs of the control group without tyrosol, bacterial and yeast cells had integrity with round or oval-shaped cell wall and membrane structures. However, some cytoplasmic damage was observed in the central parts of the bacterial cells in addition to a few, partially microbody-like vacuoles in the central parts of the yeast cells. The morphological findings in the control group were much more regular and complete than those in the treatment groups. Yeast cells appeared to interact with some bacterial cells through their wall portions, and there was a slight change in bacteria–yeast cell wall contact points, indicative of binding (Figures 3A and 3B). When exposed to tyrosol at MIC, cytoplasmic ruptures, prominent and numerous microbodies (peroxisomes), cell membrane–cytoplasm separation, damage to the walls in the form of collapse and many small vesicles were observed in yeast cells. Cell membrane–cytoplasm separation was evident in *S*. *mutans* ATCC 25175 cells. Bacteria–yeast interactions were noticeable in some areas (Figures 3C and 3D). When exposed to tyrosol at 1/2 MIC, damage to the walls in the form of collapse, cytoplasmic ruptures, and melting were observed in yeast cells, and there was cell wall cytoplasm damage in a small number of bacterial cells (Figures 3E and 3F).

**Figure 4:**
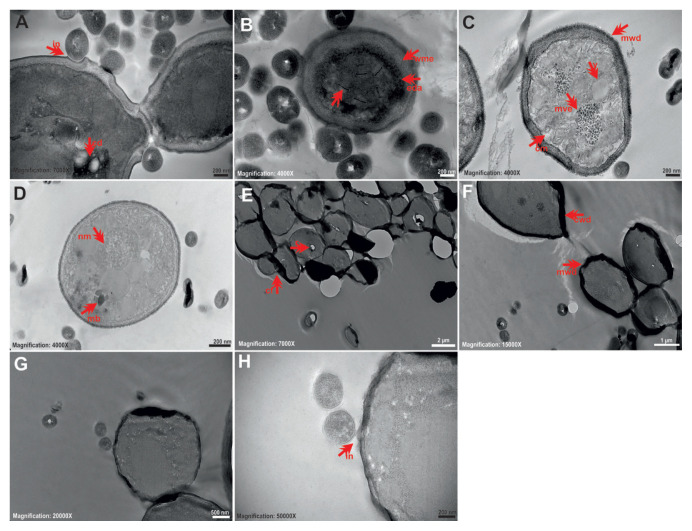
Representative TEM images showing ultrastructural alterations in dual-species planktonic cultures of *S*. *mutans* ATCC 25175 and *C*. *tropicalis* 1678 after exposure to AMB and AMP at 1/2 MIC and MIC. As a result of treatment with AMB at MIC (A, B), yeast cells had microtubule-like filamentous structures (f), cell wall–membrane expansions (wme), electron-dense cytoplasmic appearance (eda), yeast–bacteria interaction zones (in), and extensive cytoplasmic damage (cd). Upon exposure to AMB at 1/2 MIC (C, D), yeast cells displayed membrane–wall damage (mwd), numerous microvesicular structures (mve), vacuole formations (v), nuclear melting (nm), cytoplasmic disintegration (cm), and the presence of microbodies (mb) and vacuoles (v). As a result of treatment with AMP at MIC (E, F), only a few bacterial cells were observed, alongside cytoplasmic ruptures (r), cell wall damage (cwd), and fusion events (cf) in yeast cells, with membrane–wall damage (mwd) also present. In samples exposed to AMP at 1/2 MIC (G, H), relatively intact bacterial cells were seen along with prominent interaction points (in) between yeast and bacteria. Scale bars: A, B, C, D = 200 nm; E = 2 μm; F = 1 μm; G, H = 500 nm.

Figures 4A–4D show representative TEM images of ultrastructural changes in planktonic dual cultures exposed to AMB at MIC and 1/2 MIC. Microtubule-like filamentous structures were striking in the cytoplasm of yeast cells as a result of exposure to AMB at MIC. Additionally, cell wall membrane expansion, electron-dense appearance in the cytoplasm, yeast–bacteria interaction zones, and damage to the cytoplasm were observed (Figures 4A and 4B). When exposed to tyrosol at 1/2 MIC, there was membrane–wall damage, numerous microvesicular structures, vacuole formations, nuclear melting, cytoplasmic melting, microbodies (peroxisomes), and vacuole formations in yeast cells (Figures 4C and 4D). Figure 4E–4H show TEM images of ultrastructural changes of planktonic dual cultures exposed to AMP at MIC and 1/2 MIC. As a result of exposure to AMP at MIC, there were fewer bacterial cells, and cytoplasmic ruptures and cell wall damage were observed. In this group of cells, yeast were also fused and wall membrane damage was noticeable (Figures 4E and 4F). When exposed to tyrosol at 1/2 MIC, bacterial cells had a relatively healthier morphology, and yeast–bacteria interaction points were evident (Figures 4G and 4H).

### 3.4. SEM results for single yeast species biofilm

**Figure 5:**
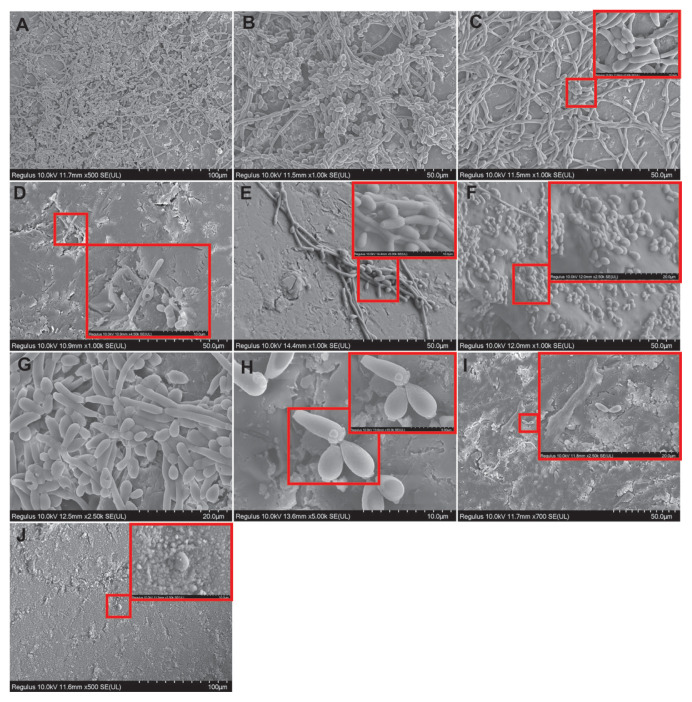
SEM images showing morphological changes in *C*. *tropicalis* 1678 cells on acrylic resin surfaces with and without tyrosol treatment. (A, B) Control group without tyrosol application. (C, D) Cells treated with tyrosol at MIC concentration before and after biofilm formation, respectively, (E, F) Cells treated with 2× MIC of tyrosol before and after biofilm formation, respectively, (G, H) Cells treated with AMB at MIC before and after biofilm formation, respectively. (I, J) Cells treated with 0.12% chlorhexidine gluconate before and after biofilm formation, respectively. Scale bars: A = 100 μm; B, C, D, E, F = 50 μm; G = 20 μm; H = 10 μm; I = 50 μm and 100 μm.

In the control group samples, the majority of *C*. *tropicalis* cells on the acrylic surface were in filamentous form and a mature biofilm structure was observed (Figures 5A and 5B). This structure consisted of a dense and heterogeneous network of yeast, pseudohyphae, and hyphae. Hyphal budding structures were also observed. The EPS structure was not clearly distinguishable, but this may have been due to the intense dehydration stages during SEM monitoring. Prebiofilm samples treated with tyrosol at the MIC had deformed and reduced biofilm structures. There were shrinkages and fusions in the hyphae structures, and bleb formations in some yeast cells (Figure 5C). In the cells exposed to tyrosol at the MIC postbiofilm formation, the biofilm was markedly reduced. The cells had shrunk due to cytosolic loss, the hyphae and biofilm structure was greatly reduced, cavities had formed in the cells, and fused cells increased (Figure 5D). When cells were exposed to tyrosol at 2× MIC prebiofilm formation, t the hyphae and pseudohyphal structures were severely damaged, fused, and disintegrated in most places. The biofilm structure reduced significantly. However, yeast cells were more structurally protected (Figure 5E). When the cells were exposed to tyrosol at 2× MIC postbiofilm formation, the hyphae structure in the biofilm was greatly reduced, and damages such as structural deteriorations, cavity formations, and cell fusion were detected in the yeast cells (Figure 5F). In the prebiofilm group samples treated with antifungal AMB at MIC, although the biofilm structure was dense, it was observed that the cells fused, deformities occurred, and cavity formations increased (Figure 5G). In the postbiofilm group, samples treated with antifungal AMB at MIC had greatly reduced cells, but the yeast cells were structurally preserved (Figure 5H). In the groups where chlorhexidine gluconate was applied, the biofilm was reduced to a great extent in the prebiofilm and postbiofilm groups, with cell shrinkage in the latter (Figures 5I and 5J, respectively).

### 3.5. SEM results for single bacterial species biofilm

**Figure 6:**
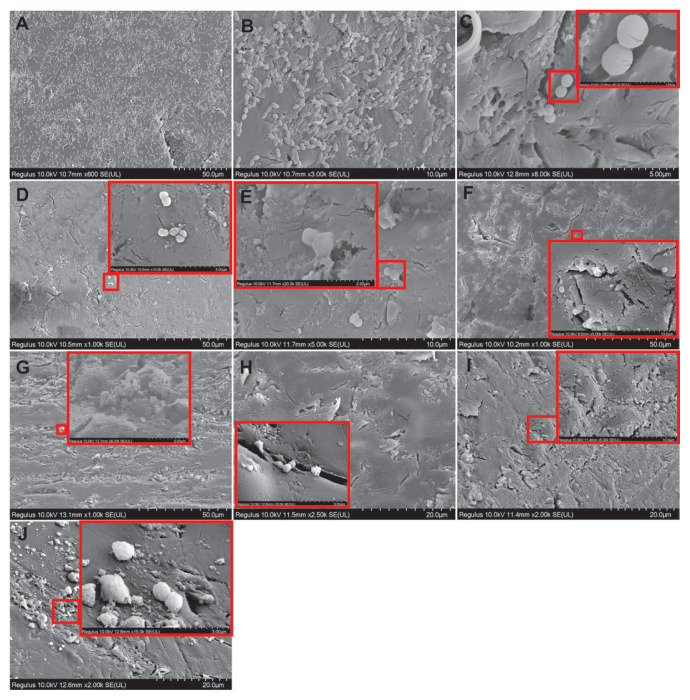
SEM images showing morphological changes in *S*. *mutans* ATCC 25175 cells on acrylic resin surfaces with and without tyrosol treatment. (A, B) Control group without tyrosol application. (C, D) Cells treated with tyrosol at MIC before and after biofilm formation, respectively. (E, F) Cells treated with 2× MIC of tyrosol before and after biofilm formation, respectively. (G, H) cells treated with AMP at MIC before and after biofilm formation, respectively; (I, J) cells treated with 0.12% chlorhexidine gluconate before and after biofilm formation, respectively. Scale bars: A = 50 μm; B = 10 μm; C = 5 μm; D = 50 μm; E = 10 μm; F, G = 50 μm; H, I, J = 20 μm.

A dense biofilm structure and cells with a typical chain structure were observed on the acrylic surface in the control samples for *S*. *mutans* ATCC 25175. The cells appeared morphologically regular (Figures 6A and 6B). When cells were exposed to tyrosol at the MIC prebiofilm formation, fewer cells were observed compared to the control. The cells that were visible were damaged (Figure 6C). When cells were exposed to tyrosol at MIC postbiofilm formation, cell numbers were significantly reduced compared to the control, and these few cells were damaged (Figure 6D). When cells were exposed to tyrosol at 2× MIC prebiofilm formation, the biofilm structure was greatly reduced and a very small number of cells observed on the surface were fused and damaged (Figure 6E). When the cells were exposed to tyrosol at 2× MIC postbiofilm formation, the biofilm was again significantly reduced and a small number of biofilm fragments were encountered (Figure 6F). The cells were almost completely reduced in prebiofilm and postbiofilm samples after treatment with the antibiotic AMP at the MIC compared to the control (Figures 6G and 6H, respectively). In a very small number of cells, there were irregularities and deformities. In the groups treated with chlorhexidine gluconate, the number of cells decreased significantly in both the prebiofilm and postbiofilm groups compared to the control group and the remaining few cells had deformities (Figures 6I and 6J, respectively).

### 3.6. SEM results for dual biofilms

**Figure 7:**
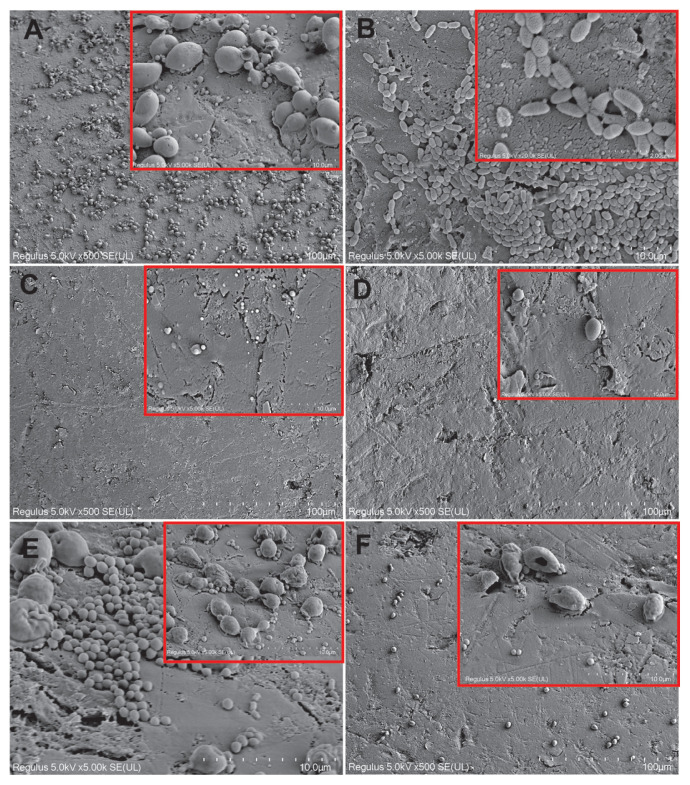
SEM images showing morphological features of dual-species (*S*. *mutans* ATCC 25175 + *C*. *tropicalis* 1678) biofilms on acrylic resin surfaces with and without tyrosol treatment. (A) Control group without tyrosol application. (B, C) Cells treated with tyrosol at MIC before and after biofilm formation, respectively. (D, E) Cells treated with 2× MIC of tyrosol before and after biofilm formation, respectively. (F) Cells treated with 0.12% chlorhexidine gluconate. Scale bars: A = 100 μm, B = 10 μm, C, D = 100 μm, E = 10 μm, F = 100 μm.

In the control samples, the cells on the acrylic surface were healthy and there was a dense biofilm on the surface (Figure 7A). When cells were exposed to tyrosol at the MIC prebiofilm formation, yeast cells were reduced and shrunken, bacteria were reduced in size, and damage was observed on the surface of cells belonging to both microorganism groups compared to the control. However, there was no significant biofilm reduction compared to the control (Figure 7B). Application of tyrosol at the MIC postbiofilm (MIC) was more effective compared to prebiofilm (MIC) application (Figure 7C). When cells were exposed to tyrosol at 2× MIC prebiofilm formation, tyrosol was highly effective and its biofilm-reducing effect was observed (Figure 7D). When the cells were exposed to tyrosol at 2× MIC postbiofilm formation, significant damage was detected in the yeasts. However, the bacteria appeared healthier and a relative decrease in the biofilm was observed in general (Figure 7E). Thus, prebiofilm application was much more effective than postbiofilm for 2× MIC application. In the groups where chlorhexidine gluconate was applied, very effective results were obtained groups compared to the control group (Figure 7F).

**Figure 8:**
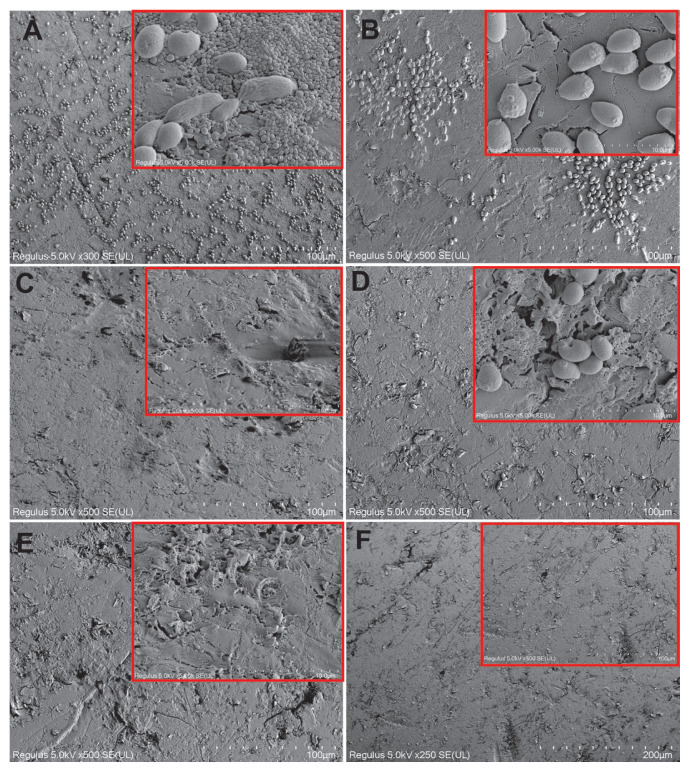
SEM images showing structural features of dual-species (*S*. *mutans* ATCC 25175 + *C*. *tropicalis* 1678) biofilms with and without AMP treatment. (A) Control group without AMP application. (B, C) Cells treated with AMP at MIC before and after biofilm formation, respectively. (D, E) Cells treated with AMP at 2× MIC before and after biofilm formation, respectively. (F) Cells treated with 0.12% chlorhexidine gluconate. Scale bars: A, B, C, D, E = 100 μm; F = 200 μm.

Figure 8 shows the effects of AMP on dual biofilms by SEM. A dense biofilm structure was observed on the acrylic surface in the control group samples. The cells were morphologically regular and have a dense biofilm appearance (Figure 8A). When cells were exposed to AMP at the MIC prebiofilm formation, there was a small decrease in biofilm with more of an effect on bacteria (Figure 8B). When cells were exposed to AMP at the MIC postbiofilm formation, the cells were almost completely reduced and AMP was very effective (Figure 8C). Thus, postbiofilm application was more effective than prebiofilm application at the MIC. When cells were treated with AMP at 2× MIC prebiofilm formation, significant biofilm reduction and occasional yeast lysis were detected (Figure 8D). Cells were very effective in samples treated with AMP at 2× MIC postbiofilm formation (Figure 8E). Postbiofilm application was more effective than prebiofilm application at 2× MIC. Chlorhexidine gluconate treatment was more effective than the control group (Figure 8F).

**Figure 9:**
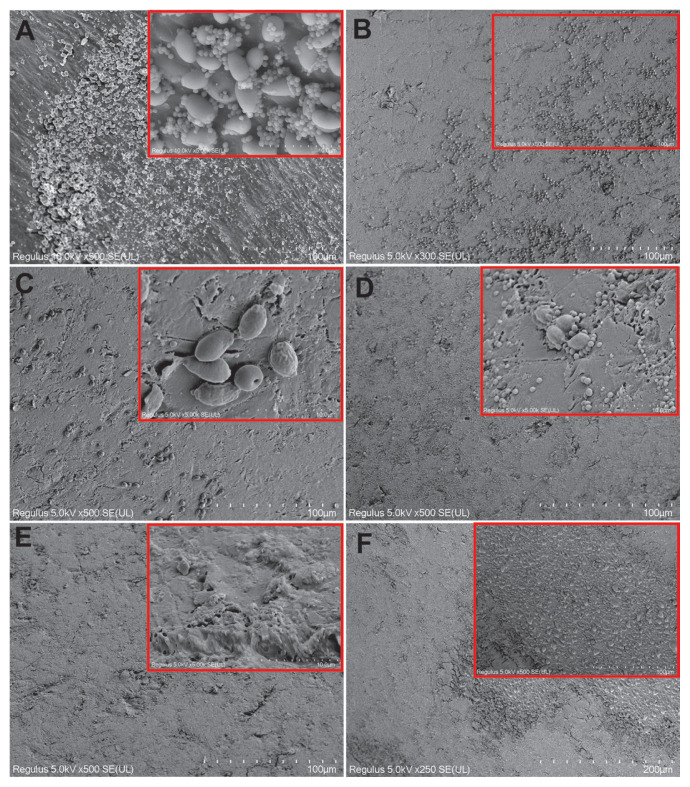
SEM images showing dual-species (*S*. *mutans* ATCC 25175 + *C*. *tropicalis* 1678) biofilm morphology with and without AMB treatment. (A) Control group without AMB. (B, C) Cells treated with AMB at MIC before and after biofilm formation, respectively. (D, E) Cells treated with AMB at 2× MIC before and after biofilm formation, respectively. (F) Cells treated with 0.12% chlorhexidine gluconate. Scale bars: A, B, C, D, E = 100 μm, F = 200 μm.

Figure 9 shows the effects of AMB on dual biofilms by SEM. A dense biofilm structure was observed on the acrylic surface in the control group samples. The cells were morphologically healthy and had extensive biofilm formation (Figure 9A). When cells were exposed to AMB at the MIC prebiofilm formation, there was a reduction in biofilm (Figure 9B). When cells were exposed to AMB at the MIC postbiofilm formation, the cells were almost completely reduced and AMB was very effective (Figure 9C). Thus, there was significant biofilm reduction in both groups. Biofilm reduction was also detected when cells were treated with AMB at a 2× MIC prebiofilm formation (Figure 9D). It was found to be very effective in samples treated with antifungal AMB at 2× MIC after cells formed biofilm (postbiofilm) (Figure 9E). Thus, postbiofilm application was more effective than prebiofilm application at 2× MIC. Chlorhexidine gluconate treatment was more effective compared to the control group (Figure 9F).

### 3.7. XTT reduction test results

Table 2 shows the XTT absorbance values observed for single-species biofilms of *C*. *tropicalis* 1678, *S*. *mutans* ATCC 25175, and the dual biofilm of the two species. *C*. *tropicalis* biofilm cells treated with tyrosol at 2× MIC had a 39% decrease in metabolic activity and a 42% decrease at the MIC compared to the control group. However, this was not statistically significant. AMB application caused a 47% decrease in 2× MIC and 48% decrease in metabolic activity at the MIC compared to the control (p < 0.05). There was a difference between the effect of tyrosol and AMB for *C*. *tropicalis* compared to the control, where MB had a stronger biofilm-reducing effect (p < 0.05). However, neither tyrosol nor AMP had a significant biofilm-reducing effect on single *S*. *mutans* biofilm cells, and there was no significant difference between the effect of tyrosol and AMP. Although the absorbance value in the dual culture increased by approximately 15% compared to the control single absorbance value of *C*. *tropicalis*, this increase was not statistically significant. Compared to the control single absorbance value of *S*. *mutans*, the dual culture absorbance value increased by more than 100% and was statistically significant (p < 0.05). When tyrosol was applied at 2× MIC in the dual culture, there was a 33% biofilm-reducing effect compared to the control. When applied at the MIC, a 2% increase in absorbance was observed, which was not statistically significant. In the dual culture, the biofilm-reducing effect of AMB was 11% at 2× MIC and 17% at the MIC compared to the control, and this was significantly lower than the effect of AMB applied alone on *C*. *tropicalis* (p < 0.05). While AMP did not have a significant biofilm-reducing effect on single *S*. *mutans*, biofilm resistance was observed in the dual culture with an increase in absorbance of over 100% at 2× MIC and 70% increase in absorbance at MIC compared to the control. When AMB and AMP were used together, there was a 24% reduction at both concentrations.

### 3.8. Determination of cultivable biofilm cells

The total number of cultivable cells on the surfaces used for single and dual biofilms was evaluated by the plate counting method after incubation. The results are presented in Table 3. All dilution rates were evaluated and the dilution rate data of 10^−5^, which was observed to be most suitable for counting, was taken as the baseline. Tyrosol increased the number of cultivable *S*. *mutans* ATCC 25175 cells, while it decreased the *C*. *tropicalis* 1678 cells at MIC and 2× MIC. In the dual culture, tyrosol slightly increased biofilm at the MIC, and reduced it at 2× MIC. The count results in both tyrosol and antibiotic/antifungal application groups were similar to the XTT data. However, the coefficient differences observed in the increase or decrease rates in the application results may be due to experimental differences.

### 3.9. Cell surface hydrophobicity test

Table 4 shows the contact angle values obtained from single and dual biofilms as a result of the treatment of active substance at 2× MIC. In *S*. *mutans* ATCC 25175, the contact angle was 28.63° in the control group, 10.8° in the tyrosol treatment group, and 36.28° in the AMP treatment group. This showed that tyrosol at 2x MIC reduced the contact angle for *S*. *mutans* and made the surface more hydrophilic, and did not show a significant reducing effect in parallel with the XTT results. Following AMP application, the surface changed from hydrophilic to hydrophobic, in line with the XTT results. In *C*. *tropicalis* 1678, the contact angles were 13.36° in the control group, 34.62° in the tyrosol treatment group, and 20.24° in the AMB treatment group. Thus, tyrosol increased the contact angle and the surface became more hydrophobic. AMB changed the surface again from hydrophilic to hydrophobic.

For the dual biofilm, the contact angles were 18.43° for the control group, 33.08° in the tyrosol treatment group, 9.37° in the AMP treatment group, 28.34° in the AMB treatment group, and 67.56° in the antifungal + antibiotic treatment group. Tyrosol application changed the surface from hydrophilic to hydrophobic, consistent with XTT results. In the AMP application, the control group changed from hydrophobic to hydrophilic, and was similar to the XTT results. In the dual group, the AMB treatment shifted the surface character from hydrophilic as observed in the dual control group to a more hydrophobic state. Notably, the combined application of AMB and AMP resulted in a strongly hydrophobic surface character compared to the dual control group.

XTT results of the same group also showed parallel findings. Thus, as the surface became more hydrophobic, the active substance was more effective. As the drug reduced the biofilm, the surface became more hydrophobic (Table 4).

### 3.10. CLSM findings

**Figure 10:**
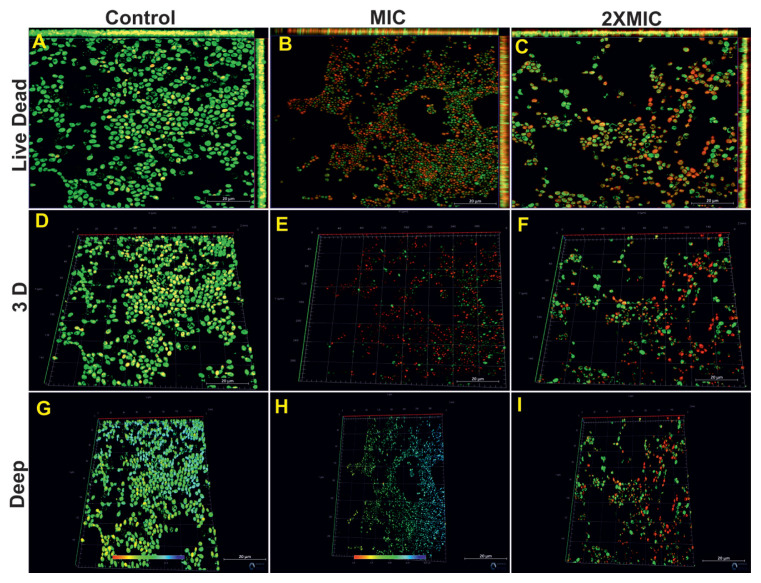
CLSM images and corresponding biofilm thickness data of *C*. *tropicalis* 1678 single-species biofilms treated with tyrosol. Two-dimensional (A, B, C) and three-dimensional (D, E, F) CLSM images, along with measured biofilm depths in micrometers (G, H, I), are presented for the control group and tyrosol treatments at MIC and 2× MIC. Live cells emit green fluorescence (SYTO 9), whereas dead cells fluoresce red (PI). Scale bars: A, B, C, D, E, F, G, H, and I = 20 μm.

In our study, a BacLight LIVE/DEAD cell viability test was used to determine whether there was cell death during fungal–bacterial biofilm development. After 48 h of incubation, live cells were determined with green fluorescent staining (Syto 9), while dead cells were determined with red fluorescent propidium iodide (PI) dye. While a dense yeast biofilm formed in the control group *C*. *tropicalis* 1678 samples, the majority of the region was dominated by green fluorescence (Figure 10A). Tyrosol application at MIC and 2× MIC significantly reduced the biofilm and dead cells were quite dense in the environment (Figures 10B and 10C). The 3D images of the same groups are shown in Figures 10D–10F. Confocal z-stack image analysis data was also used to obtain information about biofilm depth. In the control group, biofilm depth reached up to 58.40 μm (Figure 10G). Tyrosol application at MIC (39.00 μm) caused a decrease in biofilm depth. Interestingly, at 2× MIC, the biofilm depth was 52.80 μm and showed much less reduction than the control (Figures 10H and 10I).

**Figure 11:**
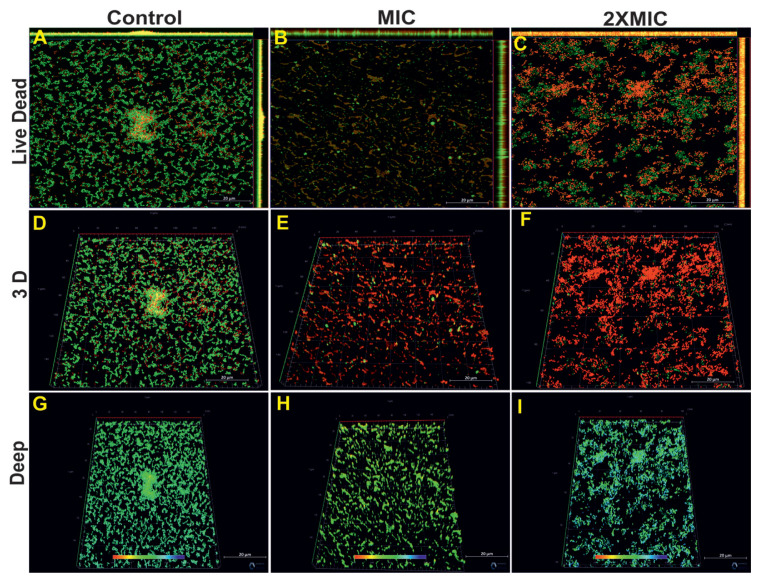
CLSM images and biofilm thickness measurements of *S*. *mutans* ATCC 25175 single-species biofilms treated with tyrosol. Two-dimensional (A, B, C) and three-dimensional (D, E, F) CLSM images, along with quantified biofilm depths in micrometers (G, H, I), are shown for the control and tyrosol treatments at MIC and 2× MIC. Live cells appear green (SYTO 9), while dead cells appear red (PI). Scale bars: A, B, C, D, E, F, G, H, and I = 20 μm.

A widespread bacterial biofilm was formed in the control group *S*. *mutans* ATCC 25175 and the majority of the region was green fluorescence (Figure 11A). There was a dense biofilm in the tyrosol application at MIC and 2× MIC, but there were also dead cells in the medium as well as live cells (Figures 11B–11C). The 3D images of the same groups are shown in Figures 11D–11F. Confocal z-stack image analysis data was also used to obtain information about biofilm depth. In the control group, biofilm depth reached up to 36.40 μm (Figure 11G). Tyrosol application did not cause a significant change in the biofilm depth at MIC (36.25 μm) and 2× MIC (32.40 μm) (Figure 11H and 11I).

**Figure 12:**
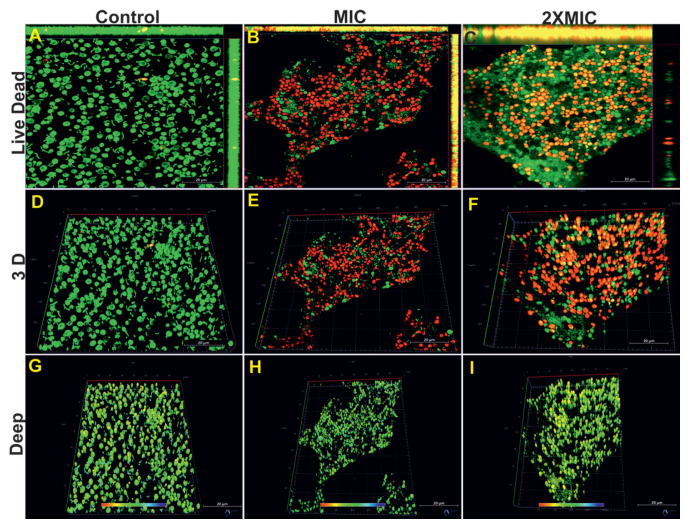
CLSM images and biofilm thickness measurements of dual-species (*C*. *tropicalis* 1678 + *S*. *mutans* ATCC 25175) biofilms treated with tyrosol. Two-dimensional (A, B, C) and three-dimensional (D, E, F) CLSM images, along with quantified biofilm depths in micrometers (G, H, I), are shown for the control and tyrosol treatments at MIC and 2× MIC. Live cells appear green (SYTO 9), while dead cells appear red (PI). Scale bars: A, B, C, D, E, F, G, H, and I = 20 μm.

While a dense yeast–bacteria biofilm formed in the dual culture control group, the majority of the region was dominated by green fluorescence (Figure 12 A). Tyrosol application at MIC and 2× MIC significantly reduced the biofilm and dead cells were found to be quite dense in the environment (Figure 12B and 12C). The 3D images of the same groups are also shown in Figure 12D–12F. When 3D images and deep images of the same group were examined with confocal z-stack image analysis, the biofilm depth reached up to 51.80 μm in the control group (Figure 12G). Tyrosol application caused a significant decrease in the biofilm depth at the MIC (33.60 μm). Interestingly, at 2× MIC, the biofilm depth was relatively high (78.40 μm) compared to the control (Figure 12H and 12I).

### 3.11. WST-1 cell viability test

In cytotoxicity test studies, tyrosol was studied in the concentration range of 20–1.0 mg/mL. Different effects were observed on 3T3 cell viability depending on the dose as a result of 24-hour tyrosol application. The inhibitory concentration (IC50) value of tyrosol at 24 h was 1.944. Tyrosol showed a dose-dependent toxic effect on cells in the concentration range of 1–4 mg/mL. At higher concentrations, it showed a cell proliferation-inducing effect compared to the control (Table S1). After 48 h of tyrosol application, different effects were observed on 3T3 cell viability, depending on the dose. The IC50 value of tyrosol at the 48th hour was 1.5783. Similar to the 24 h results, there was a significant toxic effect on the cells depending on the dose in concentration range of 1.6–4 mg/mL. At higher concentrations, it showed a cell proliferation-inducing effect compared to the control (Table S2).

## 4. Discussion

Microorganisms often exist within polymicrobial biofilms that may involve mutualistic, commensal, or antagonistic interactions. In the human body, the diversity of microbiota plays a critical role in maintaining microbial equilibrium; however, disruptions such as antibiotic exposure may lead to dominance by opportunistic species (Brown et al., 2012; Srinivasan et al., 2021). One such dual interaction is that between *Candida* spp. and *S*. *mutans*, which often form resilient, synergistic biofilms that are difficult to treat using conventional antimicrobial strategies (Ribeiro et al., 2012; Rodrigues et al., 2019). The dual biofilm formed by *C*. *tropicalis* and *S*. *mutans* is found in natural biological environments, especially in moist, nutrient-rich, and high microbial diversity natural biological environments such as the oral cavity. Other microorganisms cohabiting in this environment can have positive and negative effects on the biofilm. For example, the presence of acid-tolerant *Lactobacillus* species or *C*. *albicans* colocalizing with *C*. *tropicalis* may promote biofilm formation and stability, while the presence of antifungal metabolite-producing *Pseudomonas aeruginosa* or pH-changing *Actinomyces* species may suppress the growth or biofilm-forming capacity of the dominant species in the biofilm. The presence of carbon sources or QS molecules in the environment may also favor the development of this dual biofilm, while the presence of molecular agents such as hydrogen peroxide or high doses of farnesol may have an inhibitory effect.

This study showed the antimicrobial and antibiofilm efficacy of tyrosol against single and dual biofilms of *C*. *tropicalis* and *S*. *mutans*. The MIC findings for both *C*. *tropicalis* and *S*. *mutans* were lower than many previous reports (Monteiro et al., 2015; Tsikopoulos et al., 2019), indicating possible strain-specific sensitivity. Therefore, tyrosol showed also strong inhibitory effects on *C*. *tropicalis* and moderate effects in dual biofilm systems, although its efficacy against *S*. *mutans* alone was limited. The fact that *S*. *mutans* is more resistant to tyrosol than *C*. *tropicalis* may be due to differences in cell membrane and wall structure. Tyrosol may have affected ergosterol synthesis and membrane permeability in the *C*. *tropicalis* cell membrane, making yeast cells more sensitive. Since *S*. *mutans* does not contain ergosterol, it is less affected by compounds with membrane-targeted effects. On the other hand, *S*. *mutans* is a bacterium well adapted to acidic environment and stress conditions and has a highly dense and protective EPS structure. This adaptation includes more effective antioxidant defense systems against reactive oxygen species (ROS) and harmful compounds. *C*. *tropicalis* may have had a more limited response to oxidative stress from molecules such as tyrosol. Furthermore, tyrosol is a fungal QS molecule and affects processes such as morphogenesis, filamentation, or cell division in yeast cells. The bacterium *S*. *mutans*, on the other hand, may not contain a direct target for this molecule and may therefore be more resistant.

Structural analysis using SEM and TEM suggested widespread cellular and architectural damage, particularly at higher tyrosol concentrations. TEM images showed multitargeted cellular damage by tyrosol, including membrane rupture, cytoplasmic melting, and mitochondrial damage (Yapıcı et al., 2021). In dual cultures, yeast–bacterial interaction points were frequently observed, supporting the idea of physical cooperation in biofilm architecture (Pidwill et al., 2018; Krüger et al., 2019). The direct contact between yeast and bacterial cells plays an important role in biofilm organization, increasing the stability and structural integrity of the environment. Contact may facilitate genetic and biochemical signaling, nutrient sharing, and metabolic support. Also, virulence may increase due to changes in the level of antimicrobial resistance in the biofilm. With close contact, QS molecules can affect the gene expression of both bacterial and yeast cells. All of these mechanisms may cause treatment resistance problems. SEM analyses also confirmed concentration-dependent antibiofilm effects of tyrosol, with 2× MIC more effective in the prebiofilm phase, consistent with prior studies showing inhibition of EPS production at early stages (Monteiro et al., 2015). Postbiofilm applications, however, led to more dramatic biofilm disruption, particularly in mixed cultures. This was corroborated by XTT findings, where metabolic activity was significantly reduced in fungal populations (Arias et al., 2016; Fernandes et al., 2016). On the other hand, the dual-species model showed signs of protective bacterial dominance and physical interactions, potentially mediated by EPS or adhesion proteins.

There was a decrease in metabolic activity for *S*. *mutans* after tyrosol application, while an increase in cell count was observed in MIC concentrations using the CFU test. This finding suggests that despite being metabolically suppressed after the application of the active substance, the cells are still alive and cultivable. An alternative hypothesis is that the release of planktonic cells into the environment due to the dissolution of the biofilm structure during the CFU test may have led to an increase in cell count, but this may not have been observed in the XTT test, which is biofilm-based and measures only metabolic activity. The presence of viable but uncultivable cells (VBNC) in the environment may also be a helpful factor in understanding infections; however, this relationship between metabolic testing and VBNC viability in culture must be confirmed using methods such as ATP measurement or live/dead staining.

Our findings support *S*. *mutans* having a tolerance to tyrosol and that this increases under dual conditions. This result is consistent with reports in the literature that *S*. *mutans* can resists active agents through EPS modulation and gene regulation pathways (Senadheera et al., 2007; Nan et al., 2009). Our CFU and XTT findings indicate that *S*. *mutans* increases in a dual biofilm structure and has bacterial dominance under stress conditions. Collaborative metabolisms between species or interactions related to EPS are also thought to contribute to this resilience (Silverman et al., 2010; Ellepola et al., 2019; Lobo et al., 2019; Wu et al., 2020).

The increase in AMP resistance in the dual biofilm structure was due to the microbial interaction between the two species and the unique characteristics of the biofilm environment. Compared to biofilms formed by single species, more complex resistance mechanisms are found in synergistic dual biofilms. For example, changes in pH, oxygen, or environmental factors in the environment, or the presence of metabolically slow-growing persister cells, can reduce the effectiveness of antibiotics such as AMP. The dense and complex matrix structure of the dual-species biofilm may significantly limit antibiotic diffusion or reduce its effectiveness. Alternatively, *S*. *mutans* cells may be protected from the antibiotic effects due to their physical positioning between *C*. *tropicalis* hyphae as a result of bacteria–yeast contact. Another possible mechanism is that antibiotic resistance genes may be induced through the QS mechanism between the two species, and further in-depth studies are needed to elucidate all these complex mechanisms.

CLSM results confirmed increased cell death in tyrosol-treated samples but also showed paradoxical increases in biofilm depth in dual cultures at high concentrations. This may be due to EPS overproduction, a defensive response reported previously (Falsetta et al., 2014; Li et al., 2023).

In biofilm studies, the changes in hydrophobicity observed after antimicrobial application are closely related to the EPS composition of the biofilm. Since the EPS structure determines the adhesion, protective properties, and hydrophobicity of the biofilm, the applied agent can reduce EPS production or change its composition. When the ratio of molecules in EPS changes, the hydrophilic or hydrophobic character of the surface changes. Therefore, not only the amount of biofilm but also the changes in chemical composition should be carefully considered in the evaluation of hydrophobicity changes. In support of this, Bourroubey et al. (2024) showed that changes in hydrophobicity directly affect EPS formation and matrix structure, suggesting a dynamic interplay between surface properties and biofilm development. Our hydrophobicity tests showed increased contact angles after tyrosol treatment in several groups, suggesting altered surface adhesion and matrix composition (Chandra et al., 2005; Khoury et al., 2020; Bourroubey et al., 2024).

In our study, NIH/3T3 fibroblast cells were used to assess the cytotoxicity of tyrosol. The results showed a concentration- and time-dependent biphasic response. At concentrations up to 4 mg/mL, tyrosol suppressed cell proliferation, whereas at higher concentrations, it unexpectedly promoted cell viability. Taken together, these results suggest that tyrosol has a narrow therapeutic window, where its biological impact depends critically on concentration and exposure time. These findings imply a hormetic effect and highlight the need for further investigation to clarify the underlying mechanisms of this dual behavior. Further mechanistic studies, such as caspase activity assays and mitochondrial membrane potential analysis, are warranted to elucidate the exact pathways involved in this concentration-dependent dual response. Although tyrosol is a phenolic compound with weak antioxidant properties, it can have proliferative effects at high doses under certain conditions. In our study, the increase in cell proliferation caused by high doses of tyrosol (4.5–20 mg/mL) may have been achieved by reducing the oxidative stress level of cells in a stressful environment and promoting proliferation. On the other hand, the application of high doses of tyrosol to cells may have triggered an adaptive protective response. There are studies in the literature showing that high doses of tyrosol can have a proliferative effect on some cell lines (Zhang et al., 2019; González-Acedo et al., 2023). However, further detailed studies on this subject are needed. In the study conducted by Loru et al. (2009), the cytoprotective effects of hydroxytyrosol and tyrosol against oxidative damage were evaluated in LLC-PK1 renal epithelial cells (Loru et al., 2009). Cell viability assays were performed after 24-hour exposures to concentrations ranging from 25 to 2500 μM. While hydroxytyrosol significantly reduced cell viability at concentrations above 500 μM, tyrosol had no apparent cytotoxicity across the tested range, suggesting a more favorable safety profile. Similarly, Lee et al. (2016) reported that tyrosol inhibited the growth of KB human oral cancer cells in a time- and dose-dependent manner, indicating that its biological effects may vary depending on the cell type and experimental conditions (Lee et al., 2016).

Polymicrobial interactions have complex interspecies dynamics that significantly influence biofilm formation and development. Interspecies interactions between non-albicans *Candida* species and *S*. *mutans* and biofilm structures are attracting increasing attention, particularly due to their role in exacerbating dental caries. Currently, natural compounds, antimicrobial peptides, or nanomaterials are being extensively researched as treatment strategies. Tyrosol, like farnesol, is a QS molecule that regulates filamentation in *C*. *albicans*. While farnesol inhibits biofilm formation by facilitating the transition from the hyphal form to the yeast form, tyrosol initiates hyphal conversion in yeast but suppresses the QS system at high doses, thereby reducing biofilm formation. Other proposed mechanisms for tyrosol’s antibiofilm effects include inducing oxidative stress, suppressing virulence and adhesion genes, or directly damaging intracellular components. Tyrosol can inhibit *Candida* biofilms in a concentration-dependent manner, but this effect is particularly promising when applied before biofilm formation. At very high doses, it has toxicity. The effect of tyrosol on *S*. *mutans* works through more indirect mechanisms, such as weakening the biofilm structure, but it is ineffective on preformed biofilms. In dual-species biofilms, tyrosol is not sufficiently effective on its own compared to powerful antibiofilm agents such as farnesol or polyphenols, but its effectiveness can be increased through combined approaches. In particular, various studies have reported synergistic effects when combined with antifungal agents such as fluconazole or amphotericin. Additionally, the risk of resistance development with tyrosol is significantly lower compared to antibiotics. Natural compounds such as quercetin and resveratrol, on the other hand, can provide comprehensive antibiofilm effects at lower concentrations than tyrosol and through mechanisms such as inhibiting the QS system or suppressing virulence genes.

This study provides important insights into how tyrosol affects single and dual biofilms and planktonic cultures of *C*. *tropicalis* and *S*. *mutans*, but it is important to recognize some limitations. Despite the remarkable and promising results, one of the main limitations of the study is the small number of isolates included. On the other hand, the data obtained in vitro do not fully reflect natural biofilm ecosystems. There are many variables such as inoculation rate, incubation time, medium selection, surface type in dual biofilm studies and it is difficult to standardize these parameters. In order to support the data obtained in future research, studies in this field should be expanded with more isolates, the genes leading to resistance should be identified by molecular analysis and our data should be confirmed by in vivo experiments.

On the other hand, clinical strains of the same species can exhibit different resistance profiles in both types. While some microorganisms are resistant to the value recommended in CLSI guidelines, others can tolerate much higher concentrations. Similarly, some may be resistant to one or two antibiotics, whereas others may show resistance to several. Here, serotypic and phenotypic homogeneity differences in *S*. *mutans* affect the resistance pattern; in *C*. *tropicalis*, clonal structure, mutations, and gene expression levels can create different antifungal susceptibility profiles between strains. Therefore, strains with different resistance spectra must also be studied in detail.

As the first study to evaluate the effects of tyrosol on *C*. *tropicalis*–*S*. *mutans* biofilms, our findings highlight the potential for therapeutic application in the management of mixed oral biofilms, while underlining the need for further mechanistic and clinical research. Despite these limitations, it can be concluded that tyrosol has an inhibitory effect on oral biofilms and has the potential to be used as a natural alternative candidate for the prevention of dental caries.

## Supplementary materials

**Table S1:** Absorbance, cell survival (%) and standard deviation values obtained as a result of 24 h exposure of tyrosol to 3T3 cells at concentration ranges of 1–20 mg/mL.

Concentration (mg/mL)	OD 450 nm			Cell survival (%)	Standard deviation
20	0.509	0.515	0.528	23.522	1.450
15	0.509	0.511	0.508	22.060	0.228
10	0.475	0.454	0.455	13.285	1.768
7.5	0.488	0.430	0.447	12.127	4.451
5	0.447	0.421	0.419	7.374	2.332
4.5	0.452	0.438	0.430	9.385	1.662
4	0.398	0.407	0.399	2.316	0.736
3.5	0.400	0.419	0.426	4.814	2.008
3	0.405	0.419	0.425	5.058	1.532
2.5	0.394	0.398	0.390	0.975	0.597
2.4	0.381	0.415	0.413	2.620	2.848
2.2	0.491	0.517	0.487	20.049	2.431
2.0	0.595	0.635	0.399	41.377	3.656
1.8	0.733	0.779	0.460	67.154	4.205
1.6	0.738	0.748	0.822	69.592	6.849
1.	0.696	0.752	0.820	67.154	9.269
1.2	0.755	0.825	0.918	81.170	12.206
1.0	0.823	0.976	0.971	106.917	0.457
POZ	0.463	0.495	0.466	15.722	2.638
NEG	0.972	0.1009	0.826	100.000	4.443
BLANK	0.368	0.378	0.420	0.000	4.119

**Table S2:** Absorbance, cell survival (%) and standard deviation values obtained as a result of 48 h exposure of tyrosol to 3T3 cells at concentration ranges of 1–20 mg/mL.

Concentration (mg/mL)	OD 450 nm			Cell survival (%)	Standard deviation
20	0.468	0.468	0.453	8.013	0.680
15	0.518	0.500	0.488	11.763	1.185
10	0.477	0.453	0.440	7.404	1.474
7.5	0.439	0.415	0.428	4.583	0.943
5	0.457	0.427	0.424	5.417	1.433
4.5	0.473	0.449	0.434	6.955	1.544
4	0.117	0.408	0.409	2.772	0.048
3.5	0.409	0.430	0.433	4.263	1.027
3	0.402	0.414	0.413	2.885	0.523
2.5	0.399	0.396	0.393	1.571	0.236
2.4	0.398	0.416	0.404	2.532	0.720
2.2	0.409	0.422	0.419	3.558	0.534
2.0	0.414	0.413	0.437	4.006	1.066
1.6	0.686	0.658	0.464	21.442	9.492
1.4	0.795	0.1041	0.1026	62.869	0.721
1.2	0.904	0.944	0.965	53.654	2.433
1.0	0.1056	0.1095	0.1060	66.410	1.684
POZ	0.435	0.461	0.440	6.314	1.083
NEG	0.1440	0.1362	0.1457	100.000	3.977
BLANK	0.375	0.381	0.383	0.000	0.327

## Figures and Tables

**Table 1:** The MIC values of tyrosol against *S*. *mutans* ATCC 25175, *C*. *tropicalis* 1678, and dual culture.

Species	MICs of tyrosol	MICs of amphotericin B (μg/mL)	MICs of ampicillin (μg/mL)
*C*. *tropicalis* 1678	0.0375 (mg/mL)	1	
*S*. *mutans* ATCC 25175	2.5 (mg/mL)		1.25
Dual culture	2.5 (mg/mL)	0.125	37.5

**Note:** SD values are not shown for MIC results, as all three replicates yielded identical values (SD = 0)

**Table 2:** XTT absorbance values obtained for *S*. *mutans* ATCC 25175, *C*. *tropicalis* 1678, and dual-species biofilm.

Species	Control	Tyrosol		Amphotericin B		Ampicillin		Dual drug	

		2xMIC	MIC	2xMIC	MIC	2xMIC	MIC	2xMIC	MIC
*C*. *tropicalis* 1678	0.584±0.41	0.354±0.25	0.340±0.14	0.308±0.15	0.305±0.35				
*S*. *mutans* ATCC 25175	0.300±0.35	0.321±0.25	0.305±0.17			0.295±0.25	0.309±0.20		
dual culture	0.673±0.41	0.447±0.41	0.691±0.37	0.594±0.37	0.558±0.35	1.400±0.45	1.142±0.4	0.506±0.45	0.511±0.41

a Values for n=3

**Table 3:** Total numbers of cells that can be cultivated on the surfaces used for single and dual biofilms, obtained by the plate counting method (CFU/mL).

Microorganisms	Number of cultivable cells (CFU/mL)		

	control	tyrosol (2xMIC)	tyrosol (MIC)
*S*. *mutans* ATCC 25175	1±1 x10^5^	1.1±1 x10^6^	7±1 x10^5^
*C*. *tropicalis* 1678	2.1±2 x10^6^	1.1±1 x10^5^	1.6 ±1 x10^6^
Dual culture	1.9±1 x10^6^	3±2 x10^5^	2.1±2 x10^6^
	control	ampicillin (2xMIC)	ampicillin (MIC)

*S*. *mutans* ATCC 25175	1±1 x10^5^	3.5±2 x10^5^	1±1 x10^5^
Dual culture	1.9±2 x10^5^	2.6±2 x10^6^	2.4±2 x10^6^
	control	amphotericin B (2xMIC)	amphotericin B (MIC)

*C*. *tropicalis* 1678	1.6±1 x10^6^	3.5±1 x10^5^	5.6±2 x10^5^
Dual culture	2.1±2 x10^6^	1.4±1 x10^6^	1.1±1 x10^6^

**Table 4:** The contact angle values obtained from single and dual biofilms as a result of the treatment of active substance at 2× MIC.

Hydrophobicity test results	
Microorganism	Contact angle (°)

*S*. *mutans* Control	28.63±2.4
*S*. *mutans* Tyrosol	10.8±3.2
*S*. *mutans* Ampicillin	36.28±2.3
*C*. *tropicalis* Control	13.36±1.5
*C*. *tropicalis* Tyrosol	34.62±1.7
*C*. *tropicalis* Amphotericin B	20.24±2.4
Dual Control	18.43±2.5
Dual Tyrosol	33.08±3.2
Dual Ampicillin	9.7±3.1
Dual Amphotericin B	28.34±3.4
Dual Ampicillin + Amphotericin B	67.56±4.3
